# Low-Power Wireless Sensor Network Using Fine-Grain Control of Sensor Module Power Mode

**DOI:** 10.3390/s21093198

**Published:** 2021-05-04

**Authors:** Seongwon You, Jason K. Eshraghian, Herbert C. Iu, Kyoungrok Cho

**Affiliations:** 1 Department of Information and Communication Engineering, Chungbuk National University, Chungbuk 28644, Korea; watch@cbnu.ac.kr; 2 Electrical Engineering and Computer Science Department, University of Michigan, Ann Arbor, MI 48104, USA; 3 School of Electrical, Electronic and Computer Engineering, University of Western Australia, Perth 6009, Australia; herbert.iu@uwa.edu.au

**Keywords:** sensor node, power mode, wireless sensor networks, power management

## Abstract

Wireless sensor nodes are heavily resource-constrained due to their edge form factor, which has motivated increasing battery life through low-power techniques. This paper proposes a power management method that leads to less energy consumption in an idle state than conventional power management systems used in wireless sensor nodes. We analyze and benchmark the power consumption between Sleep, Idle, and Run modes. To reduce sensor node power consumption, we develop fine-grained power modes (FGPM) with five states which modulate energy consumption according to the sensor node’s communication status. We evaluate the proposed method on a test bench Mica2. As a result, the power consumed is 74.2% lower than that of conventional approaches. The proposed method targets the reduction of power consumption in IoT sensor modules with long sleep mode or short packet data in which most networks operate.

## 1. Introduction

In the Internet of Things (IoT), a wireless sensor network node (WSN) is a system that recognizes physical changes or signals targeting various users or environments. It is widely used in various forms for a range of purposes across home and industrial use. In general, a sensor network requires a single smart sensor node capable of detecting numerous signals such as pressure, temperature, humidity, gas flow, infrared, chemical reactions, surface, sound, steam, and others. Handling huge datasets using multiple sensory modalities is within the domain of machine learning, and interpreting the information from numerous signals is becoming increasingly important in an IoT-driven world. The common communication protocols used in WSN have been a combination of cellular and short-range wireless network technology such as Bluetooth, ZigBee, etc. [1,2]. WSN requires a long battery life, so a low-power circuit is essential with substantial resource constraints. To achieve energy efficiency, MIMO is used in the 5G environment [3]. A WSN typically has one or more connected sensors on each node and is monitoring a given physical environment with distributed multiple hops. Baniata et al. [3] used a probability sub-optimal multi-hop routing mechanism among cluster heads to increase the lifespan of the sensor network. The sensor node operates according to an event or application command, and each node communicates wirelessly. Each node has one or more sensors, a microprocessor unit, and a radio unit that receives wake-up signals. Piyare et al. [4] introduced an extension of the TSCH (Time Slotted Channel Hopping) protocol to low data rate applications using the sub-GHz frequency bands operating on TI’s System-on-Chip. They employed a special schedule for the network’s root nodes and their direct neighbors, as well as the option to have multiple root nodes in a single network. Most WSNs rely on small batteries, which is a serious bottleneck in the system [5,6]. Battery capacity is developing at a very slow rate compared to other technologies, such as integrated circuits or software design. Therefore, energy efficiency has been the prime goal when designing and deploying WSNs [7,8]. Bachir et al. address the challenges related to the reliability of communication and the efficient use of the node’s battery in WSN [9]. The performance is improved by doubling the network packet delivery ratio. Please note that the sensor node is in an idle state most of the time. David et al. [10] provided a platform wake-up receiver (WuR) with high integrability and a low cost per node to facilitate the implementation of low-cost sensor nodes. They demonstrate the feasibility of implementing a WuR with commercially available off-chip components by demonstrating a radio frequency envelope detection (RFED) WuR on a PCB mount. The most significant power consumption savings are observed when WuRs are used in low-traffic and low-density WSNs, mainly because the main transceiver is in the sleep mode for most of the time [11]. However, this type of customized platform is not cost-effective. Therefore, low-power design of circuits, architectures, algorithms, protocols, and other elements that affect power management must be carefully considered [12,13,14]. The conventional radio interface or transceiver is frequently the most power-consuming element in a WSN node, dominating both the static and dynamic power consumption of the sensor [15]. The duty cycle controlling a radio receiver and transceiver is a common and well-known solution for reducing the power consumption of WSNs. It reduces the active operation time of the sensor node but increases the wake-up time when the sensor node is in a power saving state for a long time. Therefore, in real-time communication, a wireless method with a very low duty cycle may be inadequate. Similarly, Bdiri et al. [8] introduced the wake-up receiver (WuRx), which handles idle listening while keeping the main radio completely off. The main function of WuRx is to send an interrupt signal to the processing unit when receiving a radio frequency (RF) or wake-up packet (WuPt). However, the drawback is that the WuRx must always be turned on for communication with a very short waiting time. In terms of IoT MCU, ESP32 MCU [16] is conceptually similar to the FGPM proposed in this paper, but the difference is the lack of a distinction between Idle and Active modes. Our proposed FGPM distinguishes the transmit and receive stages in specific modes, which allows for fine-grain control of the duty cycle, which has been shown to be more advantageous for power consumption control. In this paper, we propose a power management method for WSNs with five states of fine-grained power modes. We evaluate the proposed method on a test bench Mica2 [17]. As a result, of increased granularity, power consumption is reduced by 74.2% when compared to conventional methods [12]. This paper is structured as follows: Section 2 will present the communication protocol in WSNs, Section 3 will propose the fine-grained power state approach, Section 4 will present simulation results and energy consumption data of our approach, with a comparison against comparable methods, before concluding the work.

## 2. Communication in Wireless Sensor Node

### 2.1. Wireless Sensor Node Architecture

**Figure 1:**
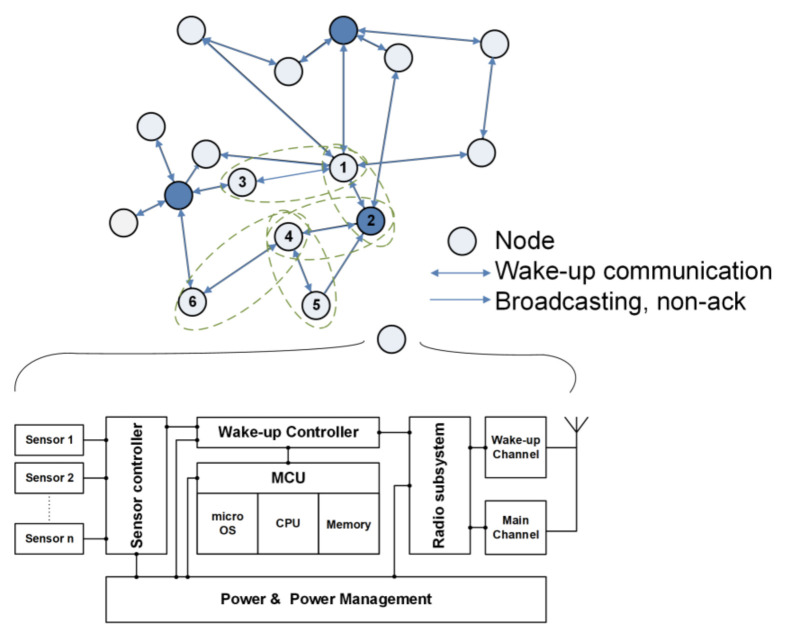
WSN Network connected multiple sensors and hardware block diagram of the sensor node module with a similar architecture to Mica2.

Each wireless sensor node has a sensing unit which detects events in the allocated area. In a given physical environment, the WSN monitors the events distributed via multi-hops routing. It communicates with the neighboring nodes to deliver the event to the user upon detection. Each inter-node communication uses a wireless transceiver in accordance with the given network protocol [18]. Figure 1 shows a basic WSN and a block diagram of a sensor module. Each node nearby events and broadcasts a signal to the users once the event is confirmed. The node module Mica2 consists of sensing units, a processor Atemga128L with memory, and an RF (CC1000) subsystem with low supply voltage, 1.6–3.6 V [17]. The Mica2’s CC1000 is a wireless data transmitter and receiver suitable for short-range communications such as gas sensors and has the advantage of easy low-power management with a simple circuit. The firmware on the microprocessor controls all sub-modules with a power management strategy.

### 2.2. Wireless Sensore Node Communication

**Figure 2:**
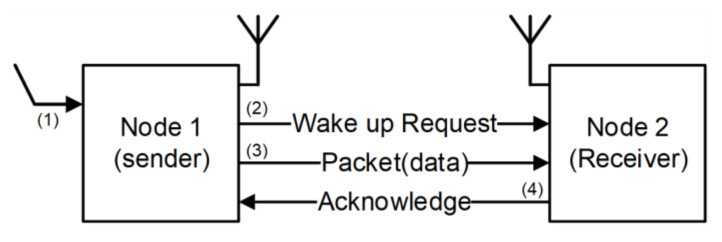
Simplified sensor node communication with a handshake protocol.

Figure 2 shows that sensor node communication is based on a handshake protocol. The first node that detects an event becomes a transmitting node (Node 1). It broadcasts a wake-up message to neighboring nodes in a fixed time slot. The node then transfers data packets to a downstream node. After packet transmission, the communication between nodes is completed by the Ack signal. When the possible communication nodes are from Node 2 to Node 4, communication is completed from the node with the highest priority (e.g., based on distance). Then, the successor node becomes a transmitting node which sends the wake-up signal to another neighboring node repeating the above procedure. When a time-out occurs due to packet loss, the scenario is restarted from the wake-up message transmission. Finally, the data on the first wake-up node is transmitted to a sink node as an alarm signal to alert the user. Additional functionalities can be integrated into the WSN as determined by the application and specifications [13]. The communication scheduling between nodes is an important issue to be considered for node power management.

**Figure 3:**
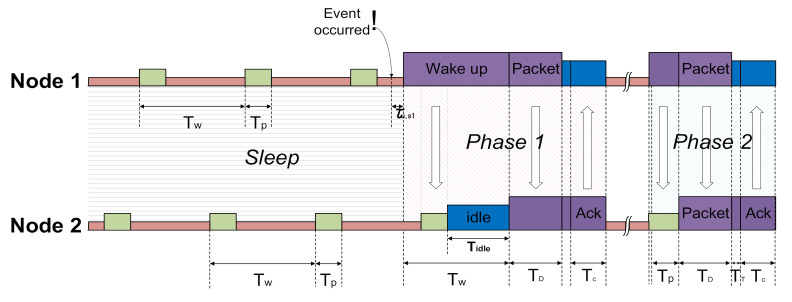
Node communication considering wake-up schedule.

Figure 3 shows the node communication schedule based on the WiseMAC protocol that determines switching from sleep mode to wake-up mode. All nodes stay in the medium idle state to receive periodically wireless wake-up signals to preserve battery life. The WiseMAC (Wireless Sensor MAC) protocol is a low-power media access control protocol designed for wireless sensor networks developed based on CSMA and preamble sampling [19]. The advantage of WiseMAC is that it dynamically reduces the size of the wake-up preamble. The wake-up signal does not require high traffic and shows that it can have low-power consumption in the wake-up state. The disadvantage is that it has a slow response performance at wake-up time from sleep. However, this trade-off is tolerable under the given circumstances, where the primary aim is to have a long battery lifetime. Table 1 shows the time parameters that are defined in Figure 3 [20]. We set the medium idle checking pulse $T_{p}\;$ such that the WSN receives a wake-up message at this time. The medium idle term $T_{W}$ is the time interval of the idle time $T_{p}$. Therefore, a node broadcasts a wake-up message during $T_{W}$ and the neighbor node detects an event at $T_{P}$. The receiver node (Node 2) returns an Ack signal during $T_{c}$ when it receives the lossless data packet. Please note that Ack includes the wake-up schedule of the receiver node as piggyback data. When the Ack is completed without any irregularities, the Node 1 transmits a wake-up message (wRx, wake-up Rx) during $T_{P}$+$T_{\mathrm{idle}}$ according to the received wake-up schedule of the Node 2. This can be applied to the WiseMAC protocol. A time-out occurs since the Ack does not normally arrive during inter-node communication. Node 1 regards it as a packet loss or wake-up failure and transmits the wake-up message and packet again in the next schedule [13,21].

## 3. Proposed Fine Grain Five States Power Mode

### 3.1. Power Management

**Figure 4:**
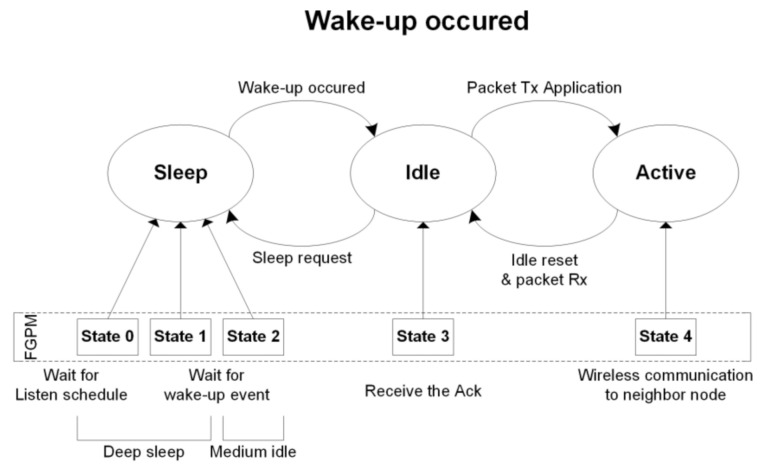
Tri-state power mode control is modified to five states power mode to reduce power consumption on WSN.

The sensor node has three operating modes: sleep, idle, and run, as shown in Figure 4. Sleep mode waits for a wake-up event, idle mode is for receiving data or standby state to receive a specific command, and run mode is for executing a specific command. In this paper, we divide the sleep mode into three modes: a deep-sleep mode (state 0), a semi-idle sensor (state 1), and a semi-idle wRx (state 2, using wake-up Rx channel). These correspond to a sleep mode which uses minimum power, a sleep mode using only the sensing unit, and a sleep mode using the wake-up Rx channel. The sleep mode transits to idle mode (s3) when a wake-up event is detected or transits to run mode (s4) according to the node schedule.

Table 2 shows modules and power consumption for each state [22,23]. As the state increases, the power consumption, as well as the time ($T_{\mathrm{TR}}$) and energy ($E_{\mathrm{TR}}$) for changing between modes also increases. Each state is classified based on the processing state of the sensor node. This power mode distinction is similar to Advanced Configuration and Power Interface (ACPI) [5,13,24]. The paper [9] also lists power consumption parameters for the preselected microcontrollers stated in the datasheet. However, it is estimated at the MCU level.

### 3.2. FGPM Operation

**Figure 5:**
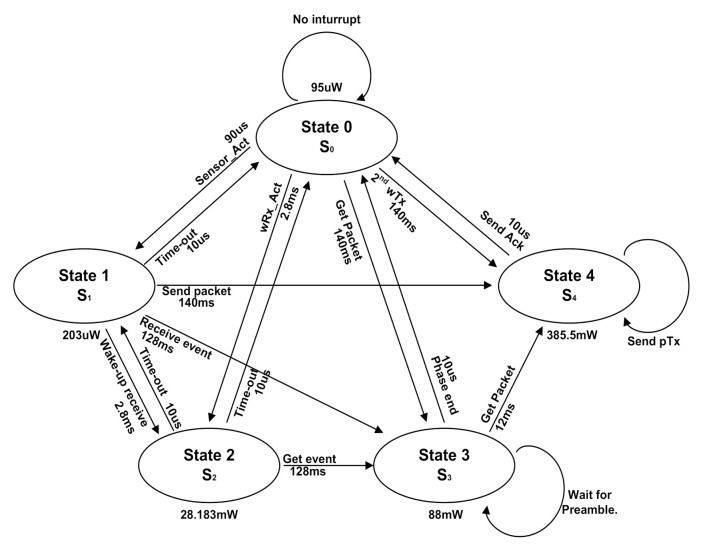
Power mode state transition for fine-grain (with five states) power control on WSN. State 0 is the initial state which is sleep state.

Figure 5 shows the transition between power modes using the proposed FGPM management technique, which controls the five states of various power modes [25]. It is represented as a finite state machine initializing at state 0. The communication schedule in Figure 3 shows how to switch between states. The sensors of the sensor module periodically switch between ON/OFF to check for events, which is the same as a state transition between State 0 and State 1. State 2 uses the wRx channel, and also periodically switches ON/OFF. This is identical to the medium idle state described above [12,20]. A node detects an event by periodically transitioning between State 0 → State 1, and State 2 → State 0 within a short period of time. When an event is detected in State 1 or State 2, it moves to State 4 through State 3. If an event is detected in State 1, then State 4 broadcasts a wake-up message and transmits the data packet. Finally, it switches to State 3 using only a packet Rx (pRx) at the receiving Ack. When an event is detected in State 2, the system switches to State 4 and returns the Ack signal. The sensor module goes back to State 0 when the scheduling of each node is completed. The inter-node communication performs the same procedure in a subsequent cycle.

### 3.3. Power Mode Control on the Sensor Node Platform

**Figure 6:**
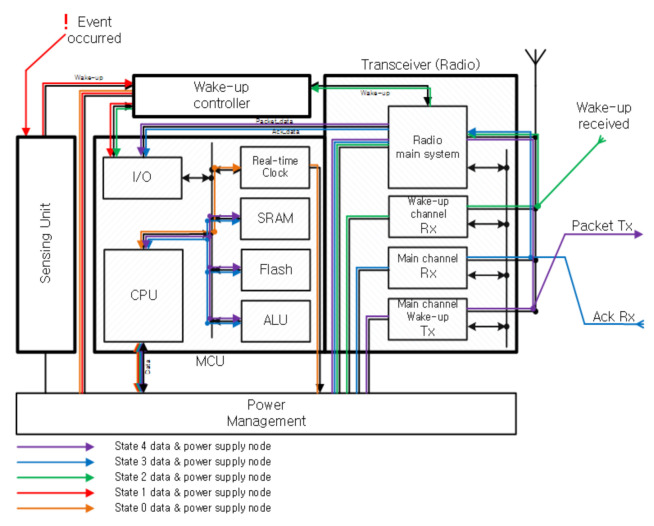
Self-waking paths on the sensor node module.

Figure 6 shows the power control of functional blocks for states of a node by using the power management procedure of the microprocessor on the module. The sensing unit and wRx communicate their signals to the MCU through the wake-up controller. By using a separate wake-up controller, the MCU can sleep while receiving those signals thus preserving the battery life for longer than using an extended standby state. The MCU controls the power management mode that it supplies, or blocks the power of each function block. Table 2 shows power consumption of each functional block for each state [26,27].

**Figure 7:**
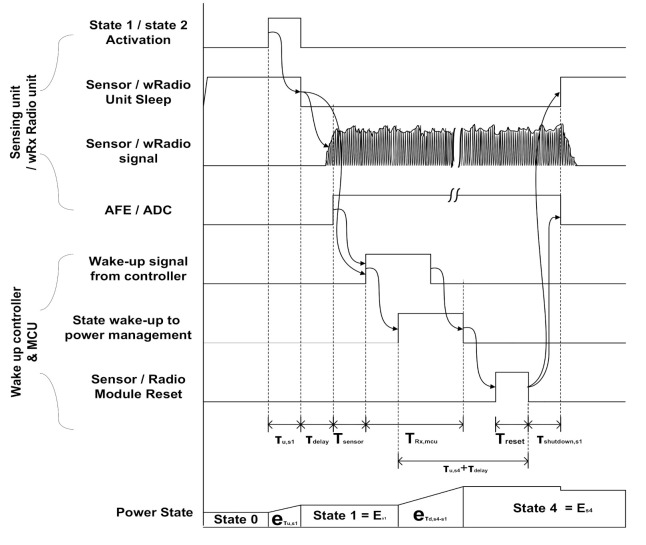
Modeling of node wake-up signal processing.

Figure 7 shows the wake-up signal process. When the sensor node switches from State 0 to State 1 and State 2 and detects an event, it transmits a wake-up message to the MCU. This enables the sensing unit and wRx channel. After the sensor node processes the signal from the sensor and antenna, they are transmitted to the wake-up controller which is the intermediate manager [28]. A controller that uses a separated power supply sends an interrupt signal to the MCU. Having completed its task, the activated MCU resets the blocks.

## 4. Energy Analysis of FGPM

### Five-State Energy Consumption

**Figure 8:**
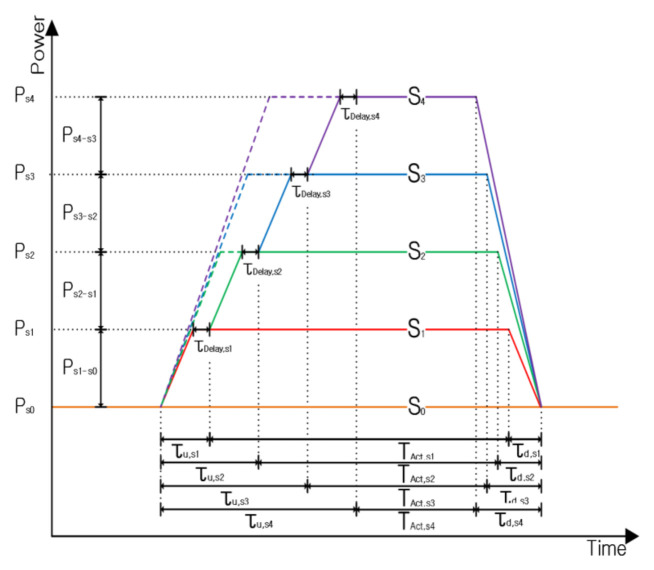
Modeling of node wake-up signal processing.

The power consumption at each state of FGPM is shown in Figure 8. When the state increases, it is transmitted through the intermediate state. As described in Section 2, node communication is processed according to a defined protocol. Even if there is a time delay, it does not affect communication when adhering to a dedicated schedule [29]. FGPM can be applied with significant savings for WSNs that have long latency and short-length data communications, such as gas sensors [13,27].

The energy consumed at a state *k* is shown in Equation (1). It is the sum of the energy consumed by the transition from State 0 to State *k*, $E_{TR},\;$ and the energy at State *k*, $E_{Act,k}$. The energy consumption, $E_{TR},\;$ while activating State *k* is equal to the product of the power consumption, $P_{k}$, and the duration of State *k*, $T_{k}$.

(1) $$E_{k}=P_{k}\times T_{k}=E_{TR}+E_{Act,k}$$

$E_{TR}$ is an important factor in the calculation of energy consumption. By increasing power mode states, the energy consumption due to state transitions also contributes to a greater power consumption. Thus, too many power modes will result in diminishing returns. The equation for $E_{TR}$ is given in Equations (2) to (7).

(2) $$E_{TR,k}=P_{TR,k}\times T_{TR,k}$$

(3) $$T_{TR,k}=\left(\;T_{s0\leftarrow k}+T_{k\leftarrow s0}\right)=\frac{\left(\tau_{u,k}+\tau_{d,k}\right)}{2}$$

where $\tau_{u,k}$ and $\tau_{d,k}$ are the time taken to activate the node in the previous State *k* − 1 and to return to the previous power reduction mode, are given as follows:

(4) $$\tau_{d,k}=\left(\;\tau_{d,k-1}+\tau_{d,\left(k-1\right)\leftarrow k}\right),\;\;\;\;\;\;\;k\geq 2$$

(5) $$\tau_{u,k}=\left(\;\tau_{u,k-1}+\tau_{delay,k}+\tau_{u,k\leftarrow \left(k-1\right)}\right),\;\;k\geq 2$$

Table 3 shows the time taken for WiseMAC protocol communication and the time required for state transitions when the node’s power consumption state is changing.

For an example, we calculate $\tau_{u,s2}$ using Equation (4), $\tau_{u,s2}=\tau_{u,s1}+\tau_{delay}+\tau_{u,s2\leftarrow s1}.$ The parameters are summarized in Table 3 as $\tau_{u,s1}=90\;\mathrm{us}$, $\tau_{u,s2\leftarrow s1}=2.8\;\mathrm{ms}$, where $\tau_{delay,k}$ is the time for the state k to stabilize (set to 0 in this experiment). Therefore, $\tau_{u,s2}=2.89\;\mathrm{ms}$. The power $P_{TR,k}$ required to change State 0 to State k can be obtained using $T_{TR,k}$. We can derive $P_{TR,k}$ as shown in Equation (6) by substituting Equation (3) into Equation (2). This is used to obtain $E_{th,k}$ in Equation (7).

(6) $$P_{TR,k}=\frac{T_{s0\leftarrow k}P_{s0\leftarrow k}+T_{k\leftarrow s0}P_{k\leftarrow s0}}{T_{TR}}=\frac{\frac{\tau_{u,k}}{2}\left|P_{k}-P_{s0}\right|+\frac{\tau_{d,k}}{2}\left|P_{k}-P_{s0}\right|}{T_{TR}}=\frac{P_{k}-P_{s0}}{\tau_{d,k}}+\frac{P_{k}-P_{s0}}{\tau_{u,k}}$$

(7) $$E_{TR,k}=\frac{P_{k}-P_{s0}}{2}\tau_{u,k}+\frac{P_{k}-P_{s0}}{2}\tau_{d,k}$$

$T_{Act,k}$ contains the time parameters $T_{W,\;}T_{p}\;\mathrm{and}\;T_{D}$ shown in Table 1, which depend on the node state. The consumed energy $E_{Act,k}$ is given in Equation (8).

(8) $$E_{Act,k}=T_{Act,k}\;\left(P_{k}-P_{s0}\right)$$

Thus, energy consumption $E_{k}$ of State *k* during $T_{k}$ is given by Equation (9).

(9) $$E_{k}\left(T_{k}\right)=P_{k}T_{Act,k}+(P_{k}-P_{s0})T_{TR}\;=P_{k}T_{Act,k}+\frac{\left(P_{k}-P_{s0}\right)}{2}\left(\tau_{d,k}+\tau_{u,k}\right)\;=P_{k}T_{Act,k}+\frac{\left(P_{k}-P_{s0}\right)}{2}\tau_{u,k}+\frac{\left(P_{k}-P_{s0}\right)}{2}\tau_{d,k}$$

Since State 4 and State 0 use the maximum and minimum power, respectively, it is clear that a lower number state will use less power. Equation (10) shows the energy saving for the case where $P_{k}\left(T_{k}\right)=P_{s4}-P_{k}$ in Equation (9) and subtracting the additional power consumption $E_{TR}$. Equation (11) shows the amount of power saving.

(10) $$E_{saved,k}\left(T_{k}\right)=\left(P_{s4}-P_{k}\right)T_{Act,k}-E_{TR}$$

(11) $$E_{saved,k}=\frac{\left(P_{s4}-P_{k}\right)T_{Act,k}-E_{TR}}{T_{k}}$$

Please note that State (*k* − *n*) (where *n* ≤ *k* − 1) uses less energy than State *k*, but absolute energy reduction is not guaranteed. To save energy at State (*k* − *n*), the energy $E_{\mathrm{TR},n}$ used in the transition from the existing State (k-n) to State *k* must be greater than the energy $E_{th,k}$ given in Equation (12).

(12) $$E_{th,n}=P_{n}\times T_{th,n}$$

We can find the minimum time $T_{th,k}$ at State k keeping $E_{save,k}>0\;$ using $P_{TR,k}$ described above is given as:

(13) $$T_{th,k-n}=\frac{1}{2}\left[\tau_{d,n}+\frac{\left(P_{k}+P_{k-n}\right)}{\left(P_{k}-P_{k-n}\right)}\right]\;\tau_{u,n}$$

## 5. Energy Consumption in Wireless Sensor Node

### 5.1. Node Energy Consumption in Sleep Mode

**Figure 9:**
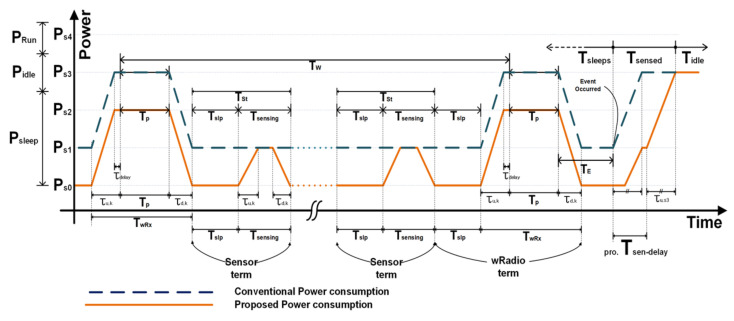
Transforming of energy states in sleep mode of a node.

The proposed FGPM has three highly granular power modes in sleep mode. In the standby state, wRadio and sensor modules consume power to detect input signals. The proposed structure repeats the use of State 0 and State 1 in order to switch the sensing unit ON/OFF. In the medium idle state, the FGPM makes a state transition from State 3 to State 2 to reduce energy consumption. This section describes the total energy consumption considering all scenarios for the state of dormancy. Table 4 shows power consumption and timing parameters for the sleep mode shown in Figure 9. $P_{k}$ is power consumption in the FGPM complying with a communication protocol. The duration $T_{k}$ for State *k* has many variables as shown in Figure 9.

Each sensor node is in a sleep state before detecting an event which is as long as i × $T_{W}$. During this time, there is one *wRx* term for sensing a wake-up *Rx* signal and the sensing time is $n=\left(T_{w}-T_{wRx}\right)/T_{st}$. Equations (14) to (16) show energy consumption while the node awaits the onset of an event.

(14) $$E_{sleeps}=\overset{j}{\underset{T_{W=1}}{\sum }}{\left(E_{wakeRx}\left(T_{P}\right)+n\cdot E_{sensor}\right)}_{T_{W}}+E_{sensed}$$

where j represents the total sleep time of a node. It is equivalent to the sum of the energy consumed from sensing, $j\times T_{W}$, and j times of energy of the medium idle state. $E_{wakeRx}$ is the energy consumption of the medium idle state of a node while receiving a wake-up signal with wireless communication. It is the largest cause for energy consumption for a node in the sleep state is given as Equation (15).

(15) $$E_{wakeRx,save}\left(T_{p}\right)=P_{s0}T_{slp}+\left(\;P_{s3}-P_{s2}\;\right)T_{P}+\left(E_{TR,s3\leftarrow s1}-E_{TR,s2}\right)$$

$E_{sensor}$ has two types of energy consumption. Based on the proposed method, $E_{sensor}∋e_{\tau_{d,s1}}=\left(P_{s1}-P_{s0}\right)\tau_{d,s1}$ because the node sleeps again if it fails to detect an event. If it detects an event, it sends an event signal to the MCU through the wake-up controller, so the energy is the same as $E_{sensor}∋e_{\tau_{u,s4\leftarrow s1}}=\left(P_{s4}-P_{s1}\right)\tau_{u,s4\leftarrow s1}$. Please note that $T_{Ack,s1}\;\geq T_{th,s1}$ can be obtained from Equation (13). Thus, the sensor energy saving is given in Equation (16).

(16) $$E_{sensor,saved}=\left(P_{s1}-P_{s0}\right)T_{slp}-\left(E_{TR,s1}+\left(\;P_{s1}-P_{s0}\;\right)\tau_{u,s1}\right)$$

Thus, the saved energy $E_{sleeps,saved}\;$ by the proposed method can be written as

(17) $$E_{sleeps,saved}=\sum_{i=1}^{\frac{T_{wait}}{T_{W}}}{\left(T_{slp}\left(n+1\right)\left(P_{s1}-P_{s0}\right)+T_{P}\left(P_{s3}-P_{s2}\right)+\left.E_{TR,s3\leftarrow s1}-\left(E_{TR,s2}+n\cdot E_{TR,s1}\right.\right)\right)}_{i}$$

### 5.2. Energy Consumption in Sleep Mode

**Figure 10:**
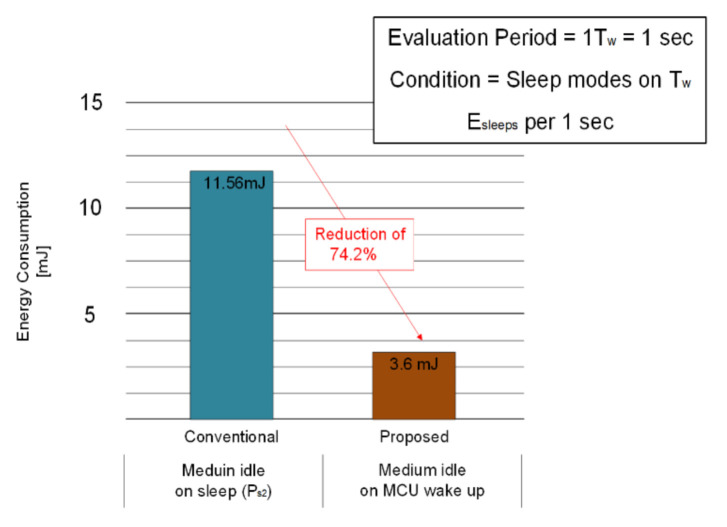
Comparison of energy consumption in the sleep mode.

Figure 10 shows energy consumption in sleep mode for both the conventional and proposed method. Based on the Mica2 node, the energy consumption when using the conventional continuous sensing unit and when the ON/OFF is repeatedly used is 178.64 uJ and 95. 96 uJ [27]. The energy saving is approximately 46.3%. Employing State 2 reduces energy consumption from 11.38 mJ to 3.5 mJ when the node is in the medium idle state, which is a 69.2% energy reduction. By adding these two factors, the Mica2 node consumes 7.96 mJ for 1 s, which results in a total energy saving of 74.2% [20,26,27].

**Figure 11:**
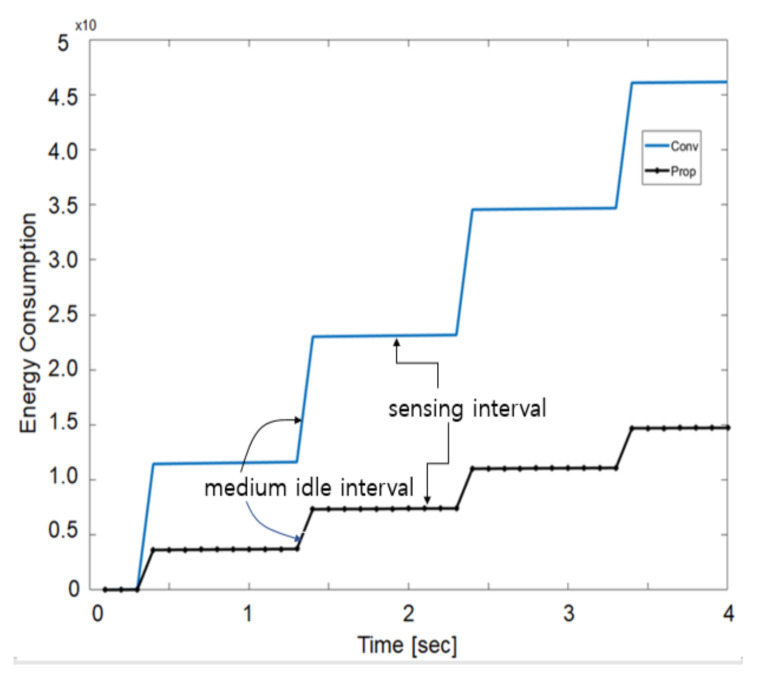
Node energy consumption in the sleep mode.

Figure 11 shows energy consumption in sleep states for 4 s to compare the existing and proposed methods. The sensing interval shows a very slow rise of energy, and the medium idle interval shows a faster rate of energy consumption increase. This is because the energy consumed by the node’s radio subsystem is large. Therefore, it can be seen that energy reduction in the radio system is emphasized. Compared with the proposed method, energy consumption varies greatly over time. Figure 11 presents a staircase energy consumption difference, but it increases linearly with respect to time.

### 5.3. Sensor Node Energy Consumption in Communication

**Figure 12:**
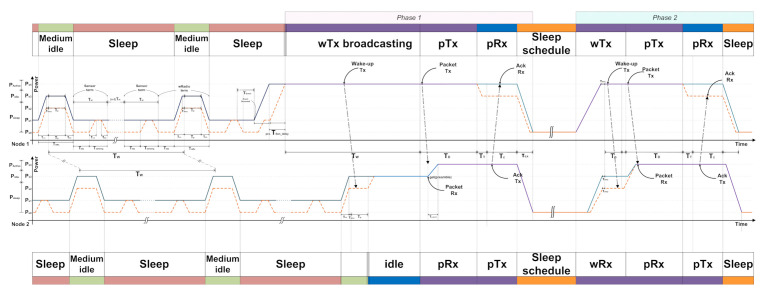
Energy state transition in WiseMAC protocol-based node communications.

In most cases, the sensor node is in the sleep state for event sensing. When a node senses an event at the sensing unit, it becomes a sender node as the first wake up node. Then, it transmits a wake-up message and data packet to the neighboring nodes and receives an acknowledge signal. In these scenarios, power consumption in a node varies with each time schedule. The WiseMAC protocol has several power consumption levels in the node’s communication scenario; however, the state of power consumption was defined only into three types: sleep, doze (idle), and communication state (RUN). The proposed approach introduces FPGM with five states, where partitioning the states enables fine-grained power management and reduced energy consumption. Figure 12 shows the change of energy consumption in communication between nodes for WiseMAC in each communication scenario. Node 1 wakes up from the sleep state by sensing an event of the sensing units and starts to communicate with Node 2. As illustrated in Figure 2, a node can have feedback after communicating at least twice for a single event. We show the advantages of the proposed fine-gained partitioned power mode that are analyzed using energy consumption benchmarks in conventional communication.

Each node sends a wake-up message and data packet to neighboring nodes. The node that first wakes up through a sensor event from the sleep mode receives an Ack. This is phase 1 communication. A time delay of $\tau_{sendelay}$ may occur due to repeated use of the sensor switching ON/OFF that uses energy, though power consumption can be reduced by using State 3 in the Rx state receiving Ack. Energy consumption is calculated in Equations (18) and (19).

(18) $$E_{conv,node1,ph1}=P_{s4}T_{W}+P_{s4}T_{D}+P_{s4}T_{T}+P_{s4}T_{c}+E_{TR,s4}$$

(19) $$E_{prop,node1,ph1}=\tau_{sendelay}P_{s1}+P_{s4}T_{W}+P_{s4}T_{D}+P_{s3}T_{T}+P_{s3}T_{c}+E_{TR,s4}$$

Nodes can communicate repeatedly even after one data packet transmission. For example, in the case of a gas sensor node, the sensor detects events periodically for a given duration, and the number of times is set by the user for checking the current physical environment and the state of the node. It is periodically fed back to the upper node, which is phase 2 communication. In phase 2, the parameter value $T_{W}$ of Node 1 is changed to $T_{P}$. Additionally, $\tau_{sendelay}$ is deleted because the medium idle state does not use the sensor. The energy consumption is given in Equations (20) and (21).

(20) $$E_{conv,node1,ph2}=P_{s4}T_{P}+P_{s4}T_{D}+P_{s4}T_{T}+P_{s3}T_{c}+E_{TR,s4}$$

(21) $$E_{prop,node1,ph2}=P_{s4}T_{P}+P_{s4}T_{D}+P_{s3}T_{T}+P_{s3}T_{c}+E_{TR,s4}$$

Node 2 wakes up from a long sleep state upon an incident wRx event. It therefore only plays the receiver role until communication with the wRx sender is completed. Node 2 becomes a receiver node that wakes up through a wireless signal without detecting an event from the sensor. In the initial communication, Node 2 can send a piggy-back Ack to inform the sending node that it is in a medium idle mode. Therefore, the energy used for the first communication of the transmitting node and the energy used in the next communication are different for m≥2  in $T_{idle,m}$, where *m* represents the number of communication events for a range of $0\leq T_{\mathrm{idle},m}\leq T_{W}-T_{P}$ [8,10,13]. Equations (18) and (19) are modified to (22) and (23).

(22) $$E_{conv,node2,ph1}=P_{s3}T_{P}+P_{s3}T_{idle,1}+P_{s4}\left(T_{D}-\tau_{u,s4\leftarrow s3}\right)+P_{s4}T_{T}+P_{s3}T_{c}+E_{TR,s4}$$

(23) $$E_{prop,node2,ph1}=P_{s2}T_{P}+P_{s3}T_{idle,1}+P_{s4}\left(T_{D}-\tau_{u,s4\leftarrow s2}\right)+P_{s4}T_{T}+P_{s3}T_{c}+E_{TR,s4}$$

From Phase 2, Node 1 can check the detailed medium idle schedule of Node 2. Thus, the time error $T_{idle}$ of $T_{P}$ approaches zero. The energy is modified to Equations (24) and (25).

(24) $$E_{conv,node2,ph2}=P_{s3}T_{P}+P_{s3}T_{idle,2}+P_{s4}\left(T_{D}-\tau_{u,s4\leftarrow s3}\right)+P_{s4}T_{T}+P_{s3}T_{c}+E_{TR,s4}$$

(25) $$E_{prop,node2,ph2}=P_{s2}T_{P}+P_{s3}T_{idle,2}+P_{s4}\left(T_{D}-\tau_{u,s4\leftarrow s2}\right)+P_{s4}T_{T}+P_{s3}T_{c}+E_{TR,s4}$$

## 6. Experimental Results

**Figure 13:**
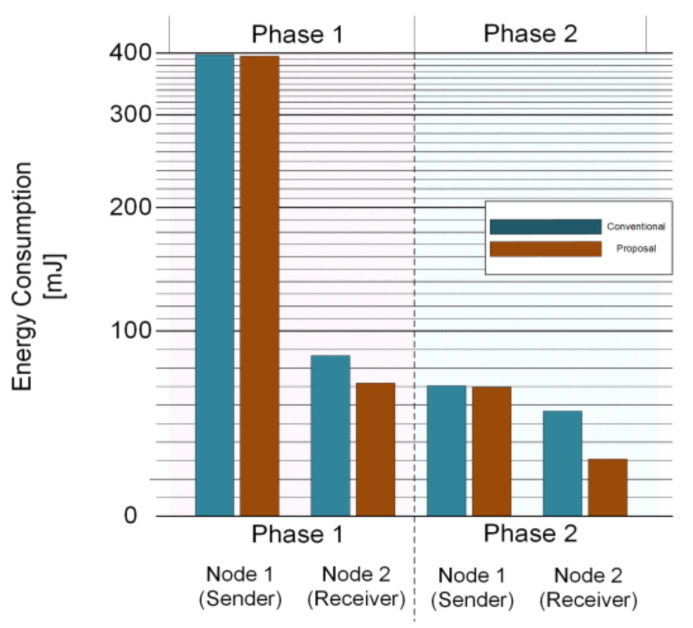
Comparison of node energy consumption for each communication scenario.

Based on the Mica2, we compared the energy consumption of the conventional and proposed approach using the Equations (18) to (25). The ad hoc network is shown in Figure 1, which accounts for inter-node communication from Node 1 to Node 6 in the network. The dotted circles are communication pairs. Node 1 wakes up Node 2 and transmits its signal. Subsequently, Node 2 wakes-up Node 3 by broadcasting a wake-up signal for other nodes. Node 2 wakes up Node 4 separately from communication between Node 1 and Node 3. In the same order, Node 4 communicates with Node 5 and Node 6. There is no communication between Nodes 3 and 4, because Node 2 has a direct line of communication with Node 4. Figure 13 shows the amount of power consumption for the single communication of a dotted circle. The time required at the send Node is $T_{W}+T_{D}+T_{T}+T_{c}+\tau_{u,s4\leftarrow s1}+\tau_{d,s4\leftarrow s1}$ , and at the receive node is $T_{W}+T_{D}+T_{T}+T_{c}+\tau_{u,s4\leftarrow s2}+\tau_{d,s4\leftarrow s1}$. However, the power consumption according to the time period is taken from Table 2. Phase 1 is for sleep mode and phase 2 is for run mode. The proposed method is not significantly different from the previous method in the sender node, but a relatively large difference can be seen in the receiver node.

**Figure 14:**
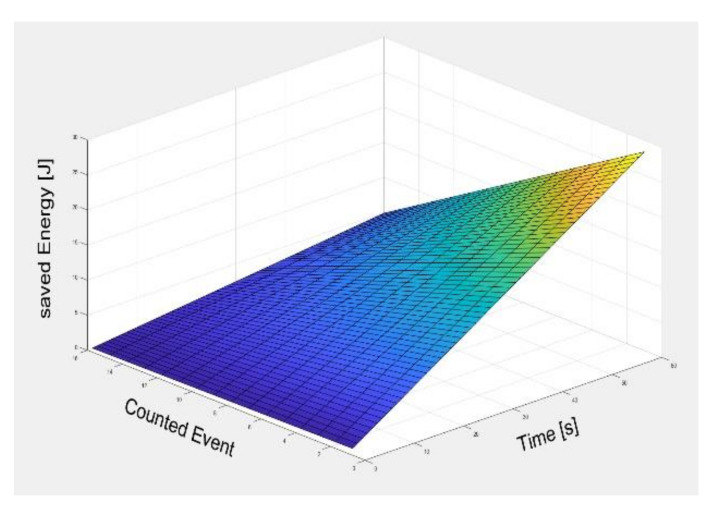
Reduced energy consumption with the parameters time and number of events.

Figure 14 shows the energy saving as a function of the parameters time and the number of events in the node. The energy consumption of the waiting time in the proposed method is about 74.2% lower than that of conventional methods. However, the energy reduction effect caused by wake-up is less than 2%, so as the number of events increases, the energy reduction of the proposed method does not necessarily scale. We assumed that the number of events occurred from a minimum 0 to a maximum 16 in a space of two minutes. There is an energy saving of about 57 J for 0 events, but about 9.7 J of energy saving is less than 15% of that for 16 events.

**Figure 15:**
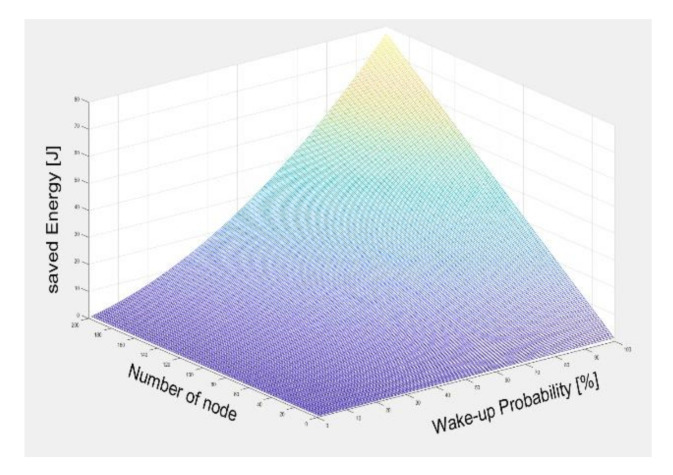
Comparison of node energy consumption for each communication scenario.

Figure 15 shows the reduced energy consumption parameter space over one minute for the number of nodes and event probability in the WSN. This shows the energy saving effect when it is assumed that each node wakes up once due to a single event. The energy saving rises as a 2nd order function for the event probability, but it is linearly increasing for the number of nodes. Based on 100 nodes, the sensor node consumes significantly less energy at 20 mJ for a 0% event probability, but energy consumption exceeds 1100 mJ at the 100% event probability. As in Figure 14, this shows that our approach can save more energy for systems of low event probability [27].

## 7. Conclusions

In this paper, we proposed a method to reduce power consumption according to the protocol usage by introducing fine-grain power modes to the WSN node. The conventional sensor system has three kinds of power modes: sleep, idle, and run. We divide the sleep mode into three states: a deep sleep mode (State 0), a medium-idle state of the sensor (State 1), and a medium-idle state of the wake-up Rx channel (State 2). Thus, the proposed WSN platform has five states of power mode, including idle and run. Even in sleep mode, power consumption is very different depending on WSN status. For example, the medium-idle mode for the sensor consumes eight times more power than the medium-idle state for wRx. We can manage the scheduling of power modes at the protocol level using a frame pending bit in the header of data packets. This power mode can be applied to the scenario of node communication, event detection, and standby according to the environment. The sleep state, which is a standby state, is an event that has the most impact on the battery life of a sensor board and has the longest time occupancy in power mode. Event recognition in sleep mode is not continuous, but periodic sensing. Thus, the minimum time to operate the sensor was calculated and we obtained the energy consumption. Besides, periodic radio signal receiving consumes the most energy in sleep mode. However, the proposed system saves energy during radio communication because the node is not used until the idle mode. The experimental results are simulations based on the architecture in Figure 1, using parameters in Table 2 and Table 4, and Figure 5. We assumed the use of gas sensors on the Mica2 platform in WSNs. The node module senses gas at state 1 of a node. The sensor node is in sleep mode and the event sensing period is set to 1 s. The proposed FGPM controls the node status of the sleep mode finely that offers energy savings of 74.2% compared to the conventional approach. This can be seen as a significant contribution to battery saving since the sensor nodes are idle in sleep mode for majority of time. However, when events occur consecutively without a sleep time, the power reduction is less than 2%. As a result, the proposed method can be expected to save power more effectively in a wireless sensor network with a low event probability or a small number of events in which most networks operate.

## Figures and Tables

**Table 1:** Node communication time parameters based on WiseMAC protocol.

Parameter	Description	Value
$T_{W}$	Medium idle term	1000 ms
$T_{D}$	Packet exchange	16 ms
$T_{c}$	Ack exchange	3.2 ms
$T_{T}$	Turnaround time between RX and TX	0.4 ms
$T_{P}$	Event sensing time	120 ms
$T_{\mathrm{idle}}$	Received wake-up and waiting for Packet receive	880 ms

**Table 2:** Power consumption of the function blocks of the sensor module.

	MCU	Memory	Sensing Unit	Radio	Wake Ctrl	Power
State 0	Sleep	sleep	off	off	off	95 uW
State 1	Sleep	sleep	on	off	on	203 uW
State 2	Sleep	on	on	wRx	on	28.2 mW
State 3	Idle	on	off	wTx, pRx, Tx	off	88 mW
State 4	Run	on	off	wTx, pRx, Tx	off	385.5 mW

**Table 3:** Power states and Time used in the sleep state.

Parameter	Value	Parameter	Value
$T_{w}$	1000 ms	$\tau_{u,s1}$	0.09 ms
$T_{D}$	16 ms	$\tau_{u,s2\leftarrow s1}$	2.8 ms
$T_{c}$	3.2 ms	$\tau_{u,s3\leftarrow s1}$	128 ms
$T_{T}$	0.4 ms	$\tau_{u,s4\leftarrow s1}$	140 ms
$T_{P}$	120 ms	$\tau_{u,s4\leftarrow s3}$	12 ms
L	1000 s	$\tau_{u,s3\leftarrow s2}$	128 ms
θ	30 ppm	$\tau_{d,s1}$	0.01 ms

**Table 4:** Power states and Time used in the sleep states.

	Scenario	Power	Time
Conv	wRadio term	$P_{s3}$, $P_{s1}$	$T_{wRx}+n\left(T_{slp}+T_{sensing}\right)$
	Medium idle	$P_{s2}$	$T_{wRx,conv}=T_{TR,s3\leftarrow s1}+T_{P}$
	Sensor term	$P_{s1}$	$T_{slp}+T_{sensing}$
Prop	wRadio term	$P_{s2}$, $P_{s0}$	$T_{wRx}+n\left(T_{slp}+T_{sensing}\right)$
	Medium idle	$P_{s2}$	$T_{wRx,prop}=T_{TR,s2}+T_{P}$
	Sensor term	$P_{s1}$, $P_{s0}$	$T_{slp}+T_{TR,s1}+T_{sensing}$

## Data Availability

Data sharing not applicable.
